# Marine-Derived Alternariol Suppresses Inflammation by Regulating T Cell Activation and Migration

**DOI:** 10.3390/md23030133

**Published:** 2025-03-19

**Authors:** Chenfeng Liu, Fudie Gu, Zhengbiao Zou, Fengli Wang, Dashuai Li, Jing Song, Yazhen Hong, Xuhui Wu, Xianwen Yang, Wen-Hsien Liu, Guangming Liu, Yu Zhou, Qingmei Liu

**Affiliations:** 1 Department of Cell Biology, School of Life Science, Anhui Medical University, Hefei 230031, Chinawfl19990316@163.com (F.W.); lds881008@163.com (D.L.); 2 Xiamen Key Laboratory of Marine Functional Food, Fujian Provincial Engineering Technology Research Center of Marine Functional Food, College of Ocean Food and Biological Engineering, Jimei University, Xiamen 361021, China; 15238875906@163.com (F.G.);; 3 Hainan Academy of Medical Sciences, Hainan Medical University, Haikou 571199, Chinayangxianwen@muhn.edu.cn (X.Y.); 4 State Key Laboratory of Cellular Stress Biology, School of Life Sciences, Faculty of Medicine and Life Sciences, Xiamen University, Xiamen 361102, China; 5 School of Public Health, Xiamen University, Xiamen 361102, China; 6 Faculty of Marine Biology, Xiamen Ocean Vocational College, Xiamen 361102, China

**Keywords:** alternariol, CD4^+^ T cells, inflammation cytokines, lung injury, cell migration

## Abstract

T cells play pivotal roles in inflammation’s initiation and progression. Exploring natural compounds that regulate T cell function is crucial for preventing and treating inflammation. Herein, we report that Alternariol (AOH), a marine-derived secondary metabolite, exerts an anti-inflammatory activity by targeting T cell function. Using an ovalbumin (OVA)-induced OT-II CD4^+^ T cell activation model, we demonstrated that AOH potently suppresses T cell proliferation and cytokine secretion, mildly promotes T cell apoptosis, and spares antigen presentation processes. Mechanistically, AOH controlled early T cell activation by inhibiting the expression of activation markers (CD69, CD25, CD44) and transcription factors (T-bet, Eomes), leading to impaired Th1 cytokine production. In vivo experiments revealed that AOH attenuated OVA-induced lung injury in mice by reducing immune cell infiltration in pulmonary tissues and draining lymph nodes. Notably, AOH dramatically suppressed OVA-specific T cells migrating to the inflammatory lung while impairing T-cell-mediated other immune cell infiltration. Collectively, AOH exhibited potent anti-inflammatory effects by modulating T cell proliferation, function, and migration, offering a promising therapeutic strategy for T-cell-mediated inflammatory diseases.

## 1. Introduction

The host immune system serves multiple functions in pathogen elimination, immune homeostasis and surveillance, and eliminating apoptotic and tumor cells [1,2]. However, persistent immune cell activation leads to acute and chronic inflammation, contributes to the pathogenesis of diverse diseases including autoimmune disorders, metabolic syndrome, and atherosclerosis [3], such as systemic lupus erythematosus (SLE), asthma, type 1 diabetes, and cardiovascular diseases [4]. Hence, precise immune modulation represents a critical therapeutic strategy for maintaining health. Inflammatory responses involve innate and adaptive immunity and their interaction. While lots of studies have evaluated the mechanisms of inflammation triggered by innate immune cells, particularly macrophage polarization and their proinflammatory roles [5,6,7], recent advances highlight the central roles of CD4^+^ T cells in the regulation of inflammation and tumor immunity [8,9]. CD4^+^ T cells exhibit plasticity in differentiation states (Th1/Th2/Th17/Treg), producing different cytokines (IFNγ, IL4, IL17, IL10) to activate or restrain immune responses. Consequently, therapeutic interventions targeting CD4^+^ T cells must balance immune competence and suppression. Current immunotherapeutics have demonstrated their clinical efficacy, including cyclosporine, and are used to treat autoimmune diseases and prevent transplant rejection by inhibiting the proliferation of T cells and the release of cytokines [10]. Alemtuzumab, a CD52^-^directed monoclonal antibody, presents the ability to deplete peripheral lymphocytes to treat relapsed or refractory lymphomas [11], and checkpoint inhibitors such as PD-1/PD-L1 antibodies enhance CD8^+^ T cell cytotoxicity to control tumors [12]. However, these drugs also show several limitations, such as hematologic toxicities (e.g., anemia) [13], hepatorenal dysfunction [14], and high costs, which restrict their broad applicability. Thus, it is still needed to search for more drugs, compounds, or natural substances to regulate T cell function, which provides potential therapeutic strategies for T-cell-related diseases.

In contrast to the land resources that have been widely used, marine ecosystems represent a largely unexplored source of bioactive compounds with unique structural diversity and therapeutic potential [15,16]. Secondary metabolites produced by marine microorganisms were identified as diverse biological activities, such as homoseongomycin, an alphavirus inhibitor isolated from Actinomycete K3-1 [17], and macrolactins derived from *Bacillus* sp. HC001 showed anti-inflammatory ability by suppressing proinflammatory cytokines and chemokines [18]. It has been reported that the secondary metabolites of *Streptomyces* sp. induce cytotoxic activity through a variety of mechanisms, including apoptosis, necrosis, inhibition of colony formation, and cell migration [19]. Despite these discoveries, the immunomodulatory potential of marine-derived compounds in T cell-mediated diseases remains largely unexplored, which limits the provision of new immunotherapy for T-cell-related inflammatory and autoimmune diseases.

In this study, using an in vitro T cell differentiation model (Patent: CN202111226760.0), we screened marine-derived compounds to evaluate the immunomodulatory activities and found alternariol (AOH), a secondary metabolite isolated from the marine fungus *Alternaria* sp., represented a potential anti-inflammatory ability by preferring to target T cell function. Previous investigations had documented AOH had biological activities in lipopolysaccharide (LPS)-induced inflammation [20], inhibiting tumor cell viability and proliferation, and other cytotoxic effects [21]. Our previous data also found AME, a structurally analogous compound of AOH, regulated mast cell degranulation to inhibit allergy [22]. However, the role of AOH in regulating T cell function remains poorly defined. To address this question, we deeply investigated the role of AOH in regulating T cell activation, proliferation, migration, cytokine production, and potential ability to treat T-cell-mediated inflammation in the lung.

## 2. Results

### 2.1. AOH Inhibits Proinflammatory Cytokine Production of T Cell In Vitro

**Figure 1:**
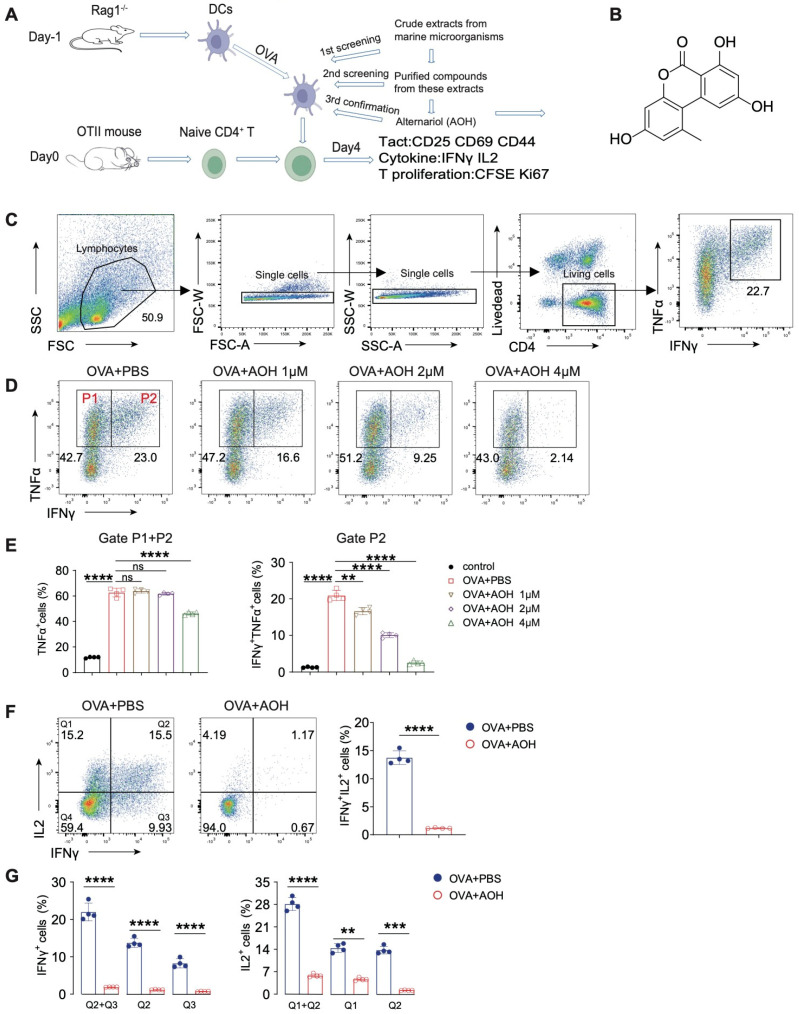
AOH inhibits proinflammatory cytokine production of T cells using OVA-OTII cell model. (**A**) Schematic diagram of the OT-II cell differentiation system. (**B**) Chemical structure of AOH. (**C**) Flow cytometric gating strategy for analyzing the cytokine released by T cells. (**D**) Flow cytometric analysis of IFNγ and TNFα secretion by T cells at varying concentrations of AOH. (**E**) Percentages of IFNγ and TNFα production. (**F**) Flow cytometric analysis of the inhibitory effects of AOH on IL-2 and IFNγ production. (**G**) Percentages of IFNγ and IL-2 released by T cells. Each symbol represents an independent biological replicate; the horizontal line represents the mean value (±s.e.m.). Statistical significance: **, *p* < 0.01; ***, *p* < 0.001; ****, *p* < 0.0001. ns, no significance.

We established an ovalbumin (OVA)-induced CD4^+^ T cell activation system (hereafter called the OVA-OTII system) to screen marine-derived extracts and compounds [23]. Using this system, we screened the numerous crude extracts derived from marine microorganisms; subsequently, we purified compounds from these extracts and identified alternariol (AOH), a secondary metabolite isolated from *Alternaria* sp., which represented an inhibitory effect in cytokine production from CD4^+^ T cells (Figure 1A). AOH was purified from the *Alternaria* sp. fermented compound, and the chemical structure was confirmed via nuclear magnetic resonance (NMR) (Figure 1B and Figure S1A–C). To delineate its immunomodulatory function, we quantified cytokine production using flow cytometry with a marked gating strategy to eliminate dead cells (Figure 1C,D). Our data revealed that greater than 50% of OVA-stimulated CD4^+^ T cells differentiated into Th1-like cells (TNFα^+^/IFNγ^+^), while AOH exhibited dose-dependent inhibition of TNFα and IFNγ production (Figure 1E). Furthermore, IL2, which is central for T cell activation, proliferation, and Th1 polarization, but AOH nearly completely suppressed IL2 and IFNγ production in CD4^+^ T cells, particularly in the Th1-polarized population (IL2^+^IFNγ^+^) (Figure 1F,G). Collecting these data, we found AOH represented an inhibitory effects for Th1-related cytokines.

### 2.2. AOH Affects T Cell Apoptosis and Inhibits T Cell Proliferation

**Figure 2:**
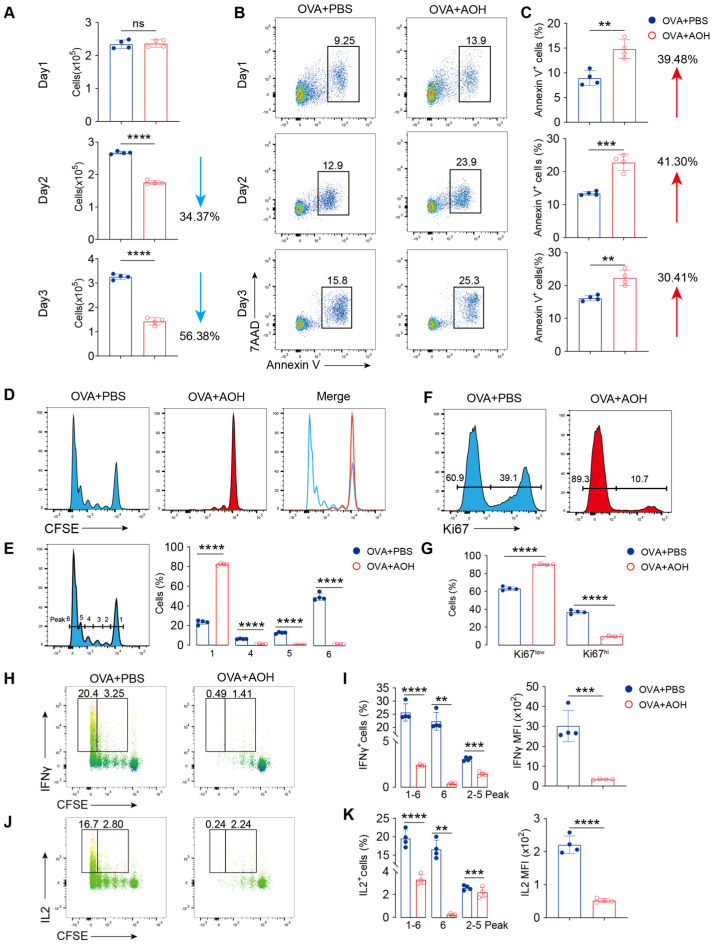
AOH modulates apoptosis and proliferation of T cells. (**A**) Dynamic changes in T cell counts after AOH treatment during 1–3 days. (**B**) Flow cytometric analysis of the effect of AOH on T cell apoptosis using Annexin V and 7AAD staining. (**C**) Apoptotic percentage of T cell mediated by AOH. (**D**) CFSE labeled cell proliferation in the presence of AOH. (**E**) Percentage of CD4^+^ T cell proliferation in 1, 4–6 generations in the presence of AOH. (**F**) Ki67 expression after AOH treatment. (**G**) Percentage of Ki67 expression in the presence of AOH. (**H**) Flow cytometric analysis of CFSE marked IFNγ expression in T cells on day 4. (**I**) Percentage and immunofluorescence intensity of IFNγ released by CD4^+^ T cells in different generations. (**J**) Flow cytometric analysis of IL-2 secretion in proliferating CD4^+^ T cells. (**K**) Percentage and immunofluorescence intensity of IL-2 released by CD4^+^ T cells in different generations. Each symbol represents an independent biological replicate; the horizontal line represents the mean value (±s.e.m.). Statistical significance: **, *p* < 0.01; ***, *p* < 0.001; ****, *p* < 0.0001. ns, no significance.

T cell differentiation and cytokine production depend on multiple factors such as cell activation, proliferation, metabolic regulation, and apoptosis [24]. Flow cytometry analysis revealed that AOH treatment significantly reduced CD4^+^ T cell numbers at days 2 and 3 after OVA stimulation (Figure 2A). Previous studies have demonstrated AOH showed an anti-tumor activity through inducing cell apoptosis [21], but little is known about the function of AOH in maintaining T cell homeostasis and survival. Using Annexin V/7AAD staining, we observed that AOH increased approximately 30% apoptotic CD4^+^ T cells after OVA stimulation, partially contributing to the reduced cell count, but could not explain near 60% cell loss (Figure 2A–C). So, we want to ask whether AOH disturbed T cell proliferation. To address this question, we employed CFSE labeling to mark the proliferated cells with individual peaks using flow cytometry to investigate the proliferative defects by AOH. The results showed that AOH-treated cells exhibited arrested proliferation, with 80% remaining in the initiated stage (peak 1), compared to 20% P1 in control groups, with 70% proliferative cells in the P4–6 stage (Figure 2D,E). Moreover, Ki-67 expression, a marker of cellular proliferation, was also markedly downregulated by AOH (Figure 2F,G). Notably, cytokine-producing cells (IFNγ^+^ or IL-2^+^) were predominantly within the proliferating population (P6), and AOH treatment abolished both their frequency and mean fluorescence intensity (MFI) expression (Figure 2H–K). Collectively, these findings showed that AOH disrupts T cell survival, proliferation, and function.

### 2.3. AOH Modulates Early T Cell Activation and Transcriptional Programming

**Figure 3:**
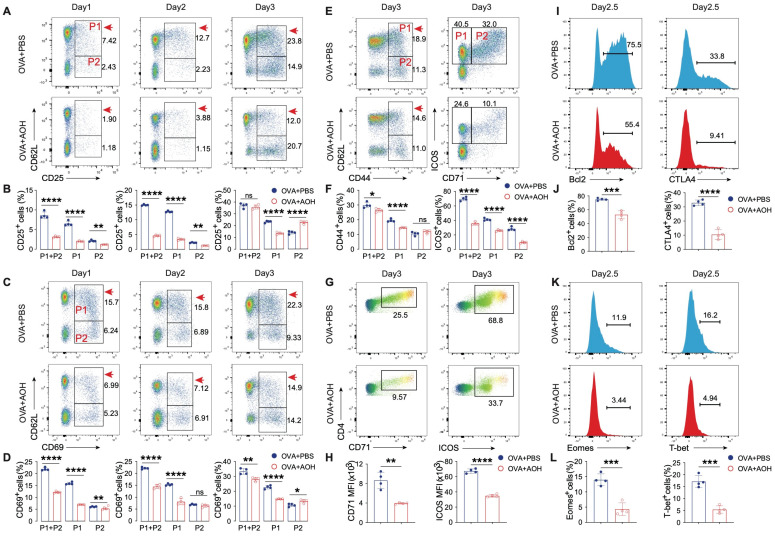
AOH modulates early T cell activation and related transcriptional programming. (**A**) Flow cytometric analysis of T cell activation using CD25 and CD62L expression, P1 (CD25^+^ CD62L^+^) and P2 (CD25^+^ CD62L^−^). (**B**) Proportions of CD25 expression in T cells. (**C**) Flow cytometric analysis of CD69 and CD62L expression in T cell activation, P1 (CD69^+^ CD62L^+^), and P2 (CD69^+^ CD62L^−^). (**D**) Percentages of CD69 expression in T cell subsets. (**E**) Flow cytometric analysis of T cell activation using CD44 and CD62L expression (**left**) and T cell differentiation using ICOS and CD71 expression (right), left P1 (CD44^+^ CD62L^+^) and left P2 (CD44^+^ CD62L^−^), right P1 (ICOS^+^ CD71^−^), right P2 (ICOS^+^ CD71^+^). (**F**) Proportions analysis of CD44 (**left**) and ICOS expression (**right**). (**G**) Flow cytometric detection of ICOS and CD71 expression. (**H**) Mean fluorescence intensity (MFI) of CD71 and ICOS in CD4^+^ T cells on day 3. (**I**) Bcl-2 (**left**) and CTLA-4 (**right**) expression in CD4^+^ T cells at day 2.5. (**J**) Quantitative analysis of Bcl-2 (**left**) and CTLA-4 (**right**) expression. (**K**) Detection of eomesodermin (Eomes) (**left**) and T-bet (**right**) expression in CD4^+^ T cells at day 2.5. (**L**) Statistical analysis of Eomes (**left**) and T-bet (**right**) expression profiles. Each symbol represents an independent biological replicate; the horizontal line represents the mean value (±s.e.m.). Statistical significance: *, *p* < 0.05; **, *p* < 0.01; ***, *p* < 0.001; ****, *p* < 0.0001. ns, no significance.

T cell activation is characterized by the upregulation of early activation markers CD69, CD25, and CD44, and the expression of differentiation-associated markers CD71 and ICOS, which are critical for T cell function. We next asked whether AOH regulated T cell activation, which caused impaired T cell proliferation and cytokine production. Activated T cells firstly enhance CD69 and CD25 expression and reduce CD62L expression, so we marked the first activated stage (P1, CD69^+^CD62L^+^, or CD25^+^CD62L^+^) and second activated stage (P2, CD69^+^CD62L^−^, or CD25^+^CD62L^−^), as shown in Figure 3A–D. Total CD69 expression (P1 + P2) was significantly reduced after AOH treatment compared to the control group at days 1 and 2, but there was no difference at day 3. Moreover, both P1 and P2 cells were decreased at days 1 and 2 after the AOH intervention, but at day 3, AOH still inhibited P1 cells but slightly enhanced the P2 percentage (Figure 3B). Notably, consideration reduced cell number after AOH treatment (Figure 2A); we also found absolutely decreased CD69^+^ T cells at days 1–3 with AOH intervention (Figure S2). CD69 is another early activated marker, and similar results were found after AOH treatment (Figure 3C,D). In contrast to early activation markers, CD44, CD71, and ICOS represent later stages of activation and differentiation. AOH treatment resulted in a marked decrease in CD44^+^ T cells, ICOS^+^ T cells, and ICOS^+^CD71^+^ double-positive populations, accompanied by reduced expression intensity of CD71 and ICOS (Figure 3E–H). Furthermore, transcriptional regulation is essential for Th1 differentiation and function, including Eomes and T-bet. Moreover, Bcl2 and CTLA4 expression are key indicators for T cell survival and activity. We found AOH controlled T cell function in a transcriptional manner by decreasing Eomes and T-bet expression. AOH also inhibited Bcl2 and CTLA4 expression, which contribute to T cell survival and prolonged activation (Figure 3I–L). Collecting these data, AOH controls the early state of T cell activation, T cell transcription, and differentiation.

### 2.4. AOH Targets Early T Cell Activation for Affecting T Cell Function Without Affecting Antigen Presentation

**Figure 4:**
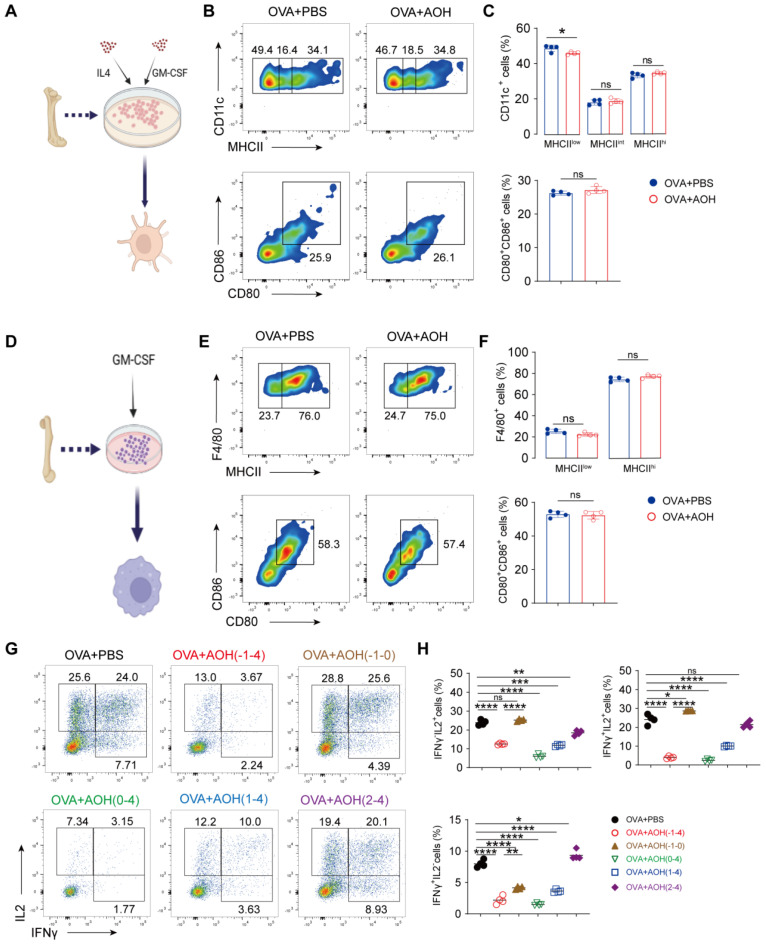
AOH is dispensable for the function of antigen-presenting cells. (**A**) Model of BMDC induction. (**B**) Flow cytometric analysis of AOH’s effects on BMDC mature and functional markers MHCII, CD80 and CD86. (**C**) Statistical analysis of MHCII, CD80 and CD86 expression. (**D**) Model of BMDM induction. (**E**) Flow cytometric analysis of AOH’s effects on BMDM mature and functional markers MHCII, CD80 and CD86. (**F**) Statistical analysis of MHCII, CD80 and CD86 expression. (**G**) Time-course analysis of AOH’s effect on inflammatory cytokine (IFNγ/IL-2) release by CD4^+^ T cells. (**H**) Percentage of IFNγ and IL-2 secreted by CD4^+^ T cells under AOH treatment. Each symbol represents an independent biological replicate; the horizontal line represents the mean value (±s.e.m.). Statistical significance: *, *p* < 0.05; **, *p* < 0.01; ***, *p* < 0.001; ****, *p* < 0.0001. ns, no significance.

T cell early activation relies on antigen-presenting cells such as dendritic cells and macrophages interacting with T cells, which present peptides via MHC-II molecules to TCRs, followed by increasing CD80 and CD86 expression, thereby driving T cell entry into an activated and functional state. To determine whether AOH modulates antigen processing, we generated bone marrow-derived dendritic cells (BMDCs) and macrophages (BMDMs) and assessed MHC-II, CD80, and CD86 expression. AOH had no impact on APC maturation or antigen presentation (Figure 4A–F), indicating a dispensable role for AOH in APC maturation and antigen-presenting function. To confirm these results, in the OVA-OTII system, we further detected the effect of AOH in DC function. AOH treatment during days 1~4 still suppressed cytokine production; however, when AOH was applied from days 1~0, which predominantly affects DC function in this time frame, cytokine production was comparable to the PBS control group (Figure 4G,H). These findings suggest that AOH preferentially targets the early T cell activation phase rather than antigen-presenting capacity by DCs.

Notably, AOH treatment during days 0~4 and 1~4 significantly inhibited IFNγ and IL-2 production, consistent with our hypothesis that AOH regulated cytokine production by preferring to target T cell early activation mechanisms. To further dissect this effect, we treated AOH at day 2~4, a period that largely bypasses early T cell activation. Under these conditions, AOH exerted a marginal effect on cytokine production (Figure 4G,H). Collectively, our data show AOH selectively regulated T cell function, preferentially targeting early stages of T cell activation to inhibit cytokine production, while exerting minimal impact on antigen presentation pathways and late-stage T cell activation.

### 2.5. AOH Alleviates OVA-Induced Pulmonary Inflammation in Mice

**Figure 5:**
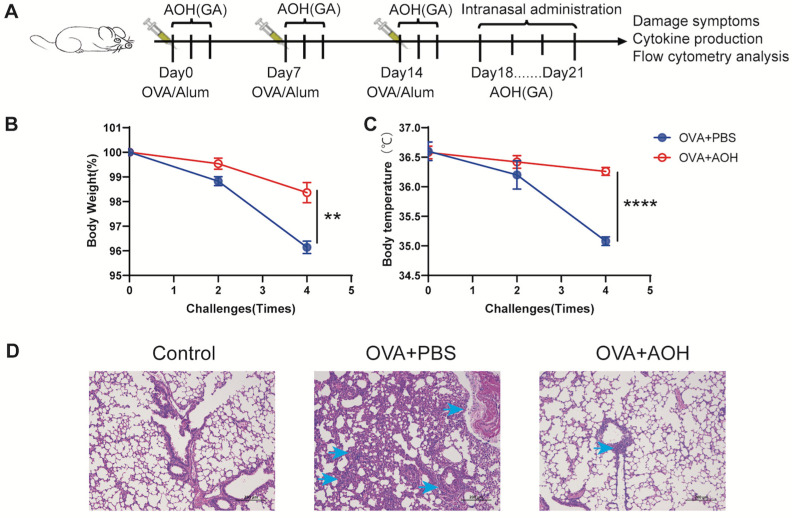
AOH attenuates OVA-induced pulmonary inflammation in mice. (**A**) A mouse model of pulmonary inflammation was established by immunization with OVA/Alum and nasal OVA. Mice were divided into two groups: OVA + PBS group and OVA + AOH group, with 5 mice in each group. AOH was gavaged in the OVA + AOH group, and the same volume of PBS was gavaged in OVA + PBS group. (**B**) Body weight changes of mice after nasal OVA administration. (**C**) Rectal temperature changes of mice after nasal OVA administration. (**D**) Histopathology changes in lung tissue injury between different groups. Each symbol represents an independent biological replicate; the horizontal line represents the mean value (±s.e.m.). Statistical significance: **, *p* < 0.01; ****, *p* < 0.0001.

Preceding data revealed that AOH suppresses T cell proliferation and cytokine production in vitro by targeting early T cell activation and transcription. Though AOH has been reported to exhibit anti-tumor activity and other biological functions in vivo, it remains unclear whether AOH regulates T cell function in vivo and its therapeutic potential in immune-related diseases. To address this issue, we employed an OVA-induced pulmonary inflammation in mice. Mice were immunized with OVA/Alum at days 0, 7, and 14, followed by intranasal OVA challenge on days 18–21. To specifically target early T cell activation, AOH was administered via gavage three times after each immunization (Figure 5A). Compared to the OVA + PBS group, AOH-treated mice exhibited improved clinical outcomes, characterized by lower weight and loss hypothermia (Figure 5B,C). OVA sensitization and re-challenge triggered aberrant T cell activation and differentiation. Pathogenic T cells migrated to the lung by OVA re-challenge and led to other immune cell infiltration in the lung, resulting in severe histopathological changes, including inflammatory solidus around the bronchioles and blood vessels, bronchiolar epithelial degeneration, and interstitial edema. AOH treatment significantly ameliorated pulmonary inflammation, as evidenced by reduced bronchiolar epithelial damage and interstitial edema, and decreased immune cell infiltration (Figure 5D). Collectively, these data represented that AOH acts as a potential therapeutic agent for treating T cell-mediated pulmonary inflammation in vivo.

### 2.6. AOH Suppresses T Cell Activation and Cytokine Production to Alleviate OVA- Induced Pulmonary Inflammation

**Figure 6:**
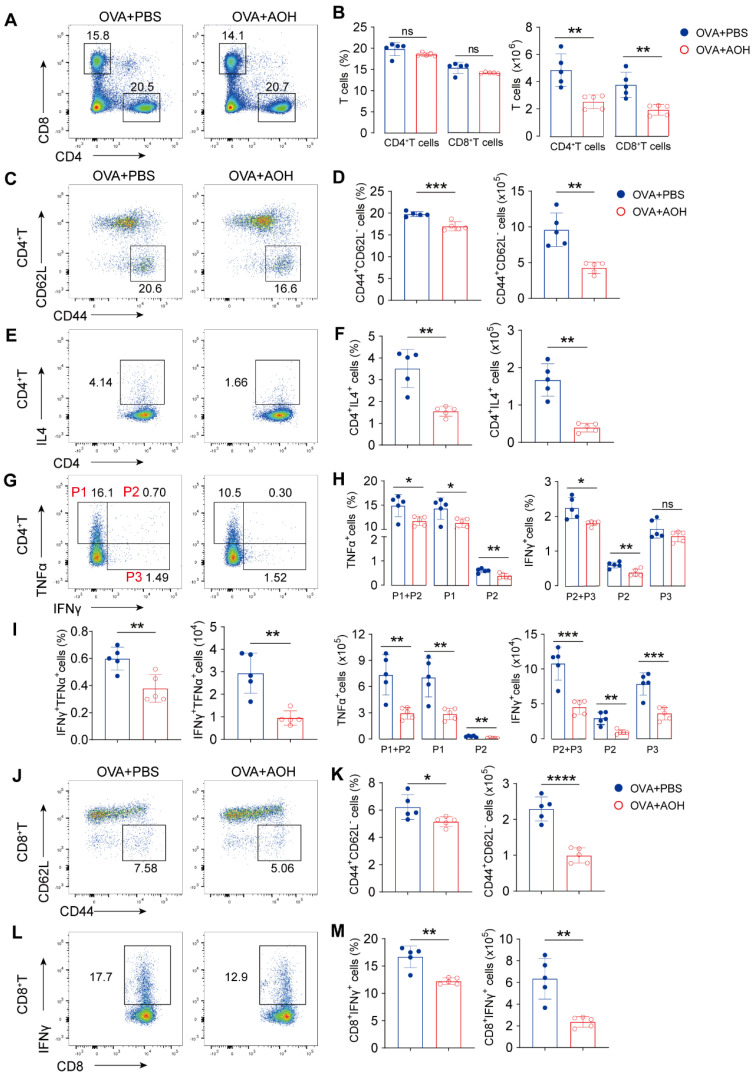
AOH controls T cell activation and cytokine production in hilar lymph nodes after lung injury. (**A**) Flow cytometric analysis of CD4^+^ T and CD8^+^ T cells in LNs. (**B**) Statistical analysis of proportions and absolute counts of CD4^+^ T and CD8^+^ T cell in LNs. (**C**) Flow cytometric analysis of CD4^+^ T cell activation by CD44^+^CD62L^-^ expression. (**D**) Statistical analysis of the proportion and counts of activated CD4^+^ T cells. (**E**) Flow cytometry analysis of IL4 production by CD4^+^ T cells. (**F**) Statistical analysis of the proportion and counts of IL-4^+^ CD4^+^ T cells in the LNs. (**G**) Flow cytometric analysis of IFNγ and TNFα expression by CD4^+^ T cells. (**H**) Statistical analysis of IFNγ and TNFα produced by CD4^+^ T cells. (**I**) Proportion and counts of IFNγ and TNFα released by CD4^+^ T cells. (**J**) Flow cytometric analysis of CD8^+^ T cell activation. (**K**) Statistical analysis of the percentage and counts of activated CD8^+^ T cells. (**L**) Cytokine of IFNγ expression produced by CD8^+^ T cells. (**M**) Statistical analysis of the proportion and counts of IFNγ released by CD8^+^ T cells. Each symbol represents an independent biological replicate; the horizontal line represents the mean value (±s.e.m.). Statistical significance: *, *p* < 0.05; **, *p* < 0.01; ***, *p* < 0.001; ****, *p* < 0.0001. ns, no significance.

To investigate whether AOH alleviated OVA-induced lung injury through regulating T-cell-mediated immune response in vivo, we first analyzed T cell populations, activation markers, and cytokine production in hilar lymph nodes after AOH treatment. The results indicated that although the percentages of CD4^+^ and CD8^+^ T cells were comparable between groups, AOH administration still resulted in a significant reduction in absolute cell numbers of CD4^+^ and CD8^+^ T cells (Figure 6A,B). Moreover, percentages and counts of activated CD4^+^ T cells (CD44^+^CD62L^−^) also decreased by AOH (Figure 6C,D). In contrast to Th1-related cytokines, OVA-induced pulmonary inflammation also recruits the Th2 population for allergic response via the Th2-IgE- mast cell/eosinophil axis. AOH treatment disrupted Th2 polarization, evidenced by reduced IL-4 secretion from CD4^+^ T cells (Figure 6 E,F). Additionally, cytokine-producing (TNFα^+^CD4^+^ T or IFNγ^+^CD4^+^ T) cells were markedly diminished, especially the dual-positive DP (TNFα^+^IFNγ^+^CD4^+^ T) subset, which is known to promote tissue inflammation and immune cell recruitment, exhibiting the pronounced reduction (Figure 6G–I). Furthermore, AOH modulated CD8^+^ T cell activation (CD44^+^CD62L^−^) and IFNγ production (Figure 6J–M), suggesting a broader impact on adaptive immune responses.

**Figure 7:**
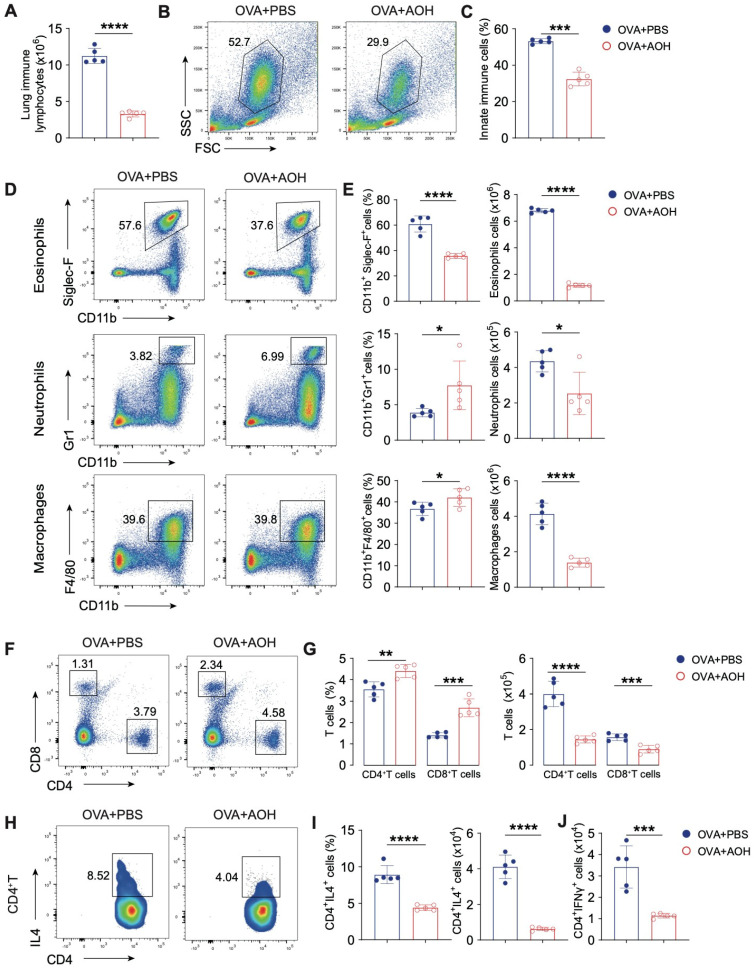
AOH inhibits immune cell infiltration and function to alleviate pulmonary inflammation. (**A**) Total immune cell counts in lung tissues. (**B**) Flow diagram for gating innate immune cell subsets (eosinophils, neutrophils, macrophages) in lung. (**C**) Statistics of absolute counts of innate immune cells in the lung. (**D**) Flow cytometric analysis of eosinophil, neutrophil, and macrophage proportions of CD45^+^ cells in lung. (**E**) Statistical analysis of the proportion and absolute counts of eosinophils, neutrophils and macrophages in lung. (**F**) Flow cytometric detection of CD4^+^ T and CD8^+^ T cell proportions. (**G**) Statistical analysis of the proportion and absolute counts of CD4^+^ T and CD8^+^ T cells in lung. (**H**) Flow cytometric analysis of IL4-secreting CD4^+^ T cells in lung. (**I**) Statistical analysis of the proportion and absolute counts of IL4 released by CD4^+^ T cells. (**J**) Statistical analysis of absolute counts of IFNγ released by CD4^+^ T cells. Each symbol represents an independent biological replicate; the horizontal line represents the mean value (±s.e.m.). Statistical significance: *, *p* < 0.05; **, *p* < 0.01; ***, *p* < 0.001; ****, *p* < 0.0001.

To investigate the immunomodulatory effects of AOH on lung inflammation, we quantified immune cell infiltration in the lung after AOH treatment. AOH administration represented a significant reduction in total immune cells in the lung (Figure 7A) and a marked attenuation of innate immune populations (size of innate immune cells is larger than adaptive immune cells, as shown in Figure 7B,C), including eosinophils, macrophages, and neutrophils (Figure 7D,E). Notably, while the relative percentage of neutrophils was slightly elevated, their absolute counts remained significantly lower in the AOH-treated group compared to PBS controls. Dramatically decreased innate immune cells disturbed the immune landscape in the lung, resulting in increased CD4^+^ and CD8^+^ T cell frequencies; however, the absolute numbers of these adaptive immune cells were concurrently diminished, consistent with suppression of IFNγ and IL-4 production by CD4^+^ T cells in the lung after AOH administration (Figure 7F–J). Collectively, these data demonstrate AOH suppressed pathogenic immune responses in pulmonary inflammation.

Previous studies reported AOH-induced cell toxicity via mitochondrial damage and enhanced an embryotoxic and immunotoxin risk in vivo. Consideration of the concerns, whether AOH dampened immune organ homeostasis, immune cell development, and survival in vivo, needs to be explored in detail. AOH was administrated to WT mice without any other stimulation for 14 days, then the spleen, lymph nodes, and thymus were analyzed, and no difference was found in PBS or AOH treatment, with similar results of cell numbers of thymocytes, splenocytes, lymph node cells, and normal T cell development, B cell development, and cytokines produced by CD4^+^T and CD8^+^T cells (Figure S3). Taken together, AOH inhibited T cell activation and cytokine production and showed an expected immuno-safety in vivo.

We previously indicated that AOH selectively targets the early phase of T cell activation to suppress proliferation and cytokine production, accompanied by fewer inhibitory effects on T cell function with AOH treatment at a later stage (Figure 4G,H). To confirm the mechanisms in vivo, as shown in Figure S4A, we treated AOH in the late stage after OVA/Alum immunization and then detected the inhibitory effect on T cell function in LNs and lung; the results showed no significant difference in T cell numbers and cytokine production in LNs and lungs (Figure S4). These results confirmed again that AOH regulated the early stage of T cell activation to inhibit T cell function in vivo and in vitro.

### 2.7. AOH Inhibits T Cell Migration to the Lung

**Figure 8:**
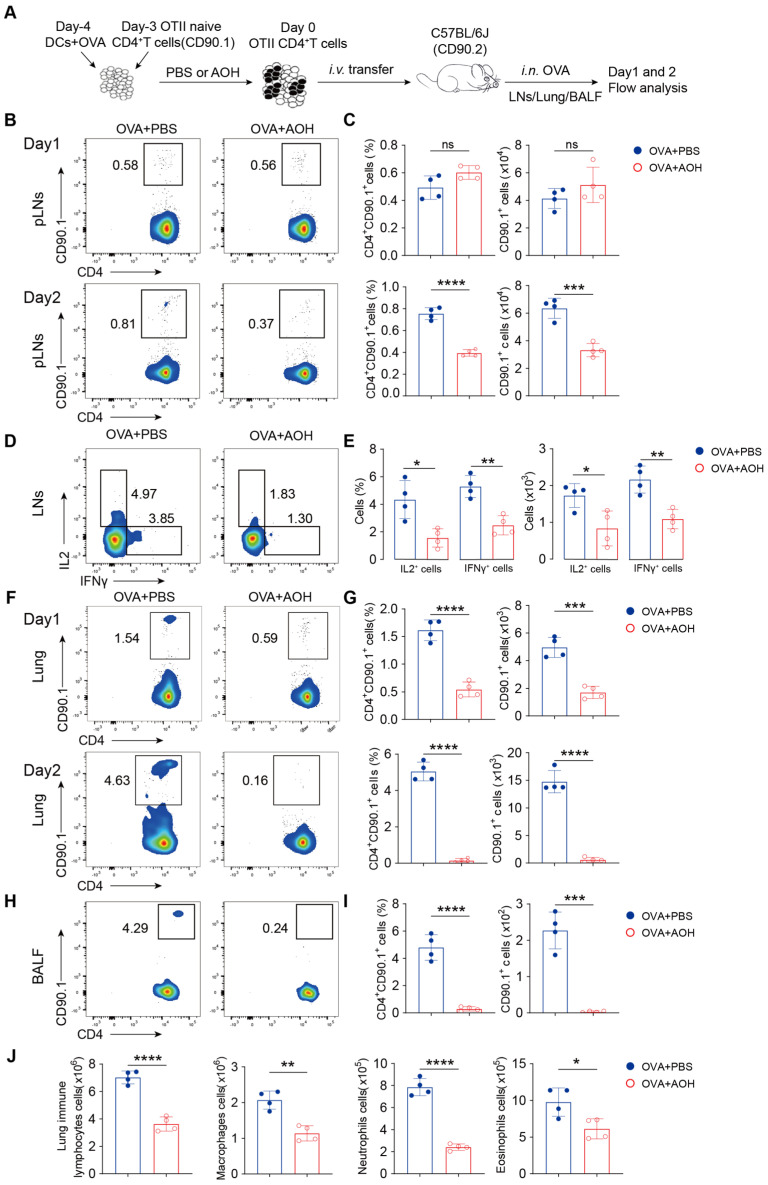
AOH regulates CD4^+^ T cell migration in vivo. (**A**) Experimental model diagram. (**B**) Quantitative analysis of CD4^+^ CD90.1^+^ T cells in peripheral lymph nodes(pLNs) on day 1 and 2. (**C**) Proportion and absolute counts of CD4^+^ CD90.1^+^ T cells in pLNs at day 1 and 2. (**D**) IFNγ and IL-2 release by CD4^+^ CD90.1^+^ T cells in LNs. (**E**) Proportions and absolute counts of IFNγ and IL-2 in pLNs. (**F**) Flow cytometric analysis of CD90.1^+^CD4^+^ T cells in lungs on days 1 and 2. (**G**) Proportions and cell numbers of CD90.1^+^ CD4^+^ T cell in lungs on days 1 and 2. (**H**) Flow cytometric analysis of CD4^+^ CD90.1^+^ cells in BALF. (**I**) Proportions and absolute counts of CD4^+^ CD90.1^+^ cells in BALF. (**J**) Absolute counts of total immune cell in lung, and macrophages, neutrophils, and eosinophils in lung. Each symbol represents an independent biological replicate; the horizontal line represents the mean value (±s.e.m.). Statistical significance: *, *p* < 0.01; **, *p* < 0.01; ***, *p* < 0.001; ****, *p* < 0.0001. ns, no significance.

In the OVA-induced pulmonary inflammation, dramatically decreased immune cell infiltration in the lung led us to hypothesize that AOH may regulate T cell trafficking to the lung. To test this question, we isolated activated OTII CD4^+^ T cells (CD90.1 background) with or without AOH pretreatment in vitro under OVA stimulation, adoptively transferred these cells into WT mice (CD90.2 background), and intranasally challenged the recipients with OVA. LNs, infiltrated immune cells in the lung and bronchoalveolar lavage fluid (BALF) were analyzed at days 1 and 2 after OVA sensitization by flow cytometry (Figure 8A). Though CD90.1^+^CD4^+^ T cells were a normal frequency in LNs at day 1, the proportion and numbers were significantly diminished by day 2 in AOH-treated mice (Figure 8B,C). Consistent with previous results, IFNγ and IL-2 production by OTII CD4^+^ T cells was also reduced following AOH administration (Figure 8D,E). Strikingly, the infiltrated numbers of OTII CD90.1^+^CD4^+^ T cells in the lung were profoundly decreased at both day 1 and day 2 under pre-treatment by AOH (Figure 8F,G), with a corresponding reduction of OTII CD90.1^+^CD4^+^ T cells in BALF at day 2 (Figure 8H,I). These data indicated that AOH affected CD4^+^ T cell homeostasis and migration to the lung, and the combined results contributed to inhibiting T cell function. Furthermore, the decrease in total lung immune cells, including macrophages, neutrophils, and eosinophils (Figure 8J), supporting AOH, inhibited the pulmonary inflammation by regulating T cell activation, cytokine production, and migration. Collectively, these results demonstrated that AOH exerted its therapeutic effects by selectively targeting the early stages of T cell activation and migration, thereby reducing T cell function and pulmonary inflammation.

## 3. Discussion

In this study, we identified AOH, a secondary metabolite derived from marine microorganisms, which potently inhibited T cell-derived inflammatory cytokines (e.g., TNFα, IFNγ) through modulating early T cell activation and migration pathways and represents a potential application for treatment of pulmonary inflammation. Currently, there is a paucity of drugs specifically targeting T cell function for treating inflammatory disorders. Cyclosporine was employed to manage autoimmune diseases and organ transplant rejection through inhibiting lymphocyte proliferation. Meanwhile, corticosteroids like dexamethasone and prednisone remained the cornerstone for controlling acute/chronic inflammation, yet long-term use of cyclosporine and corticosteroids was associated with significant adverse effects, including immunosuppression, organ toxicity (liver/kidney), and metabolic disturbances [10]. Emerging immune checkpoint inhibitors, such as pembrolizumab (Keytruda), have optimized cancer therapy through robust anti-tumor immunity but often induce extra effects like immune hepatitis or thyroiditis [25]. In contrast, AOH had shown efficacy in modulating T-cell-related inflammation while inducing tumor cell apoptosis. Combining AOH with anti-PD1 agents, which may enhance anti-tumor responses while mitigating immune-related adverse events through complementary mechanisms. Our results not only highlighted the therapeutic potential of AOH in T-cell-mediated inflammatory diseases but also underscored the value of marine microbial resources for drug discovery.

Inflammation represents a dynamic interaction and complex immune landscape between innate immune cells (e.g., macrophages, DCs) and adaptive immune cells (e.g., T cells, B cells), which is difficult to recapitulate in vitro. Relying on immortalized cell lines or simple primary cell stimulations, current in vitro models fail to capture the precise immune responses, including antigen processing, immune cell trafficking, and cytokine secretion. To address this issue, we previously reported a T cell activation and differentiation model (Patent: CN202111226760.0), represented multi-immune processes including processing and presentation by innate immune cells, as well as the activation, differentiation, and cytokine by T cells, providing a high-quality platform for screening bioactive compounds. Using the T cell differentiation model, our previous study revealed that the sulfated oligosaccharide of Gracilaria lemaneiformis (GLSO) could selectively target T cells, inhibit their activation and IFNγ production, and thereby modulate Th1 cell-mediated immunity [23]. In this study, AOH was also identified as the anti-inflammatory function by targeting T cell activation. Moreover, using this OVA-OTII system, our data revealed AOH treatment enhanced regulatory T cells (Tregs) cell differentiation in vitro and in vivo (Figure S5). Tregs are a specialized subset of CD4^+^ T cells critical for maintaining immune homeostasis, preventing autoimmunity, and modulating immune responses [26]. These results highlighted the multi-function of our OVA-OTII system during exploring T cell differentiation. Consideration of the ocean harbors a wide range of naturally active substances with diverse structures; this model could be utilized to evaluate other natural products for their anti-inflammatory effects or immunomodulatory activities.

Restraining T cell activation controls the onset and progression of T-cell-related inflammation. Zhou et al. reported a JNK pathway-associated phosphatase controls inflammatory bowel disease by suppressing CD4^+^ T cell activation [27]. A marine-derived compound, salinosporamide A, exhibited inhibitory activity on T cell activation, showing potential for the prevention and control of autoimmune diseases [28]. Prolonged T cell activation leads to sustained inflammation [29], tissue damage [30], and susceptibility to secondary infections [31]. Under the influence of chemokines, activated T cells migrate from lymphoid organs to the site of inflammation [32,33]. In addition to controlling T cell activation, regulating T cell migration can effectively mitigate inflammation. Yang et al. reported that a non-peptide CCR5 antagonist could regulate T cell migration to inhibit collagen-induced joint inflammation [34]. The AOH compound, screened in this study for its anti-inflammatory effect on T cells, was a type of mycotoxin. AOH had been noted for modulating inflammatory responses by altering macrophage morphology, disrupting cytokine signaling, and inhibiting LPS-induced immune reactions, which are involved in nuclear factor κB activation [20,35,36]. Our findings indicated that AOH could inhibit early T cell activation without disrupting normal antigen presentation. The early intervention of an overactive immune system is very beneficial for alleviating the pathological damage caused by viral infections or inflammation [37,38]. This study also found that early intervention with AOH in the lung injury model was able to inhibit T cell activation, differentiation, and migration, as well as reduce the recruitment of other cells to the site of inflammation, showing promise as a novel immunomodulator for inflammation-related disorders, warranting further preclinical investigation.

Therefore, our findings demonstrate that AOH exerted anti-inflammatory effects through an inhibition of early T cell activation, migration, and cytokine secretion, while our study identified limitations in the scope of disease models. Beyond verifying the anti-inflammatory efficacy of AOH in lung inflammation, we still need to focus on the pharmacological effects of AOH in more disease models, such as chronic inflammatory disorders (e.g., chronic immune enteritis), which are involved in sustained T cell differentiation and unbalance of Th17/Treg cells. Moreover, it was worth exploring whether AOH controlled immune response in tumors, while current evidence demonstrated AOH’s efficacy in inducing tumor cell apoptosis [19]. In addition, although we evaluated that AOH did not influence immune homeostasis, the immunotoxicity and non-immunotoxicity of AOH still need to be further studied, such as whether AOH induced toxicity and damage to endothelial or epithelial cells and whether long-term administration or higher doses of AOH caused immune suppression, gastrointestinal reactions, and liver and kidney damage. Finally, the precise molecular mechanisms underlying AOH’s inhibition of early T cell activation and migration necessitated further investigation, which will help us to provide a combination intervention of AOH with other immunomodulatory drugs to better control inflammation or with immune checkpoint inhibitors to robustly better anti-tumor effects.

## 4. Materials and Methods

### 4.1. Alternariol Extraction, Isolation, and Purification

Alternariol was obtained by culture and fermentation using *Alternaria* sp. The fungus strain (*Alternaria* sp.) was isolated from the deep-sea gammarid shrimp collected by Shanghai Rainbowfish Ocean Science and Technology at the depth of 10,870 m of the central sea of Papua New Guinea. Alternariol was obtained by culture and fermentation using *Alternaria* sp. The specimen of the fungus was preserved in the China Typical Cultures Collection Center (Wuhan, China) with the registration number of CCTCC NO: M 2022501. *Alternaria* sp. The voucher strain was preserved at the Marine Culture Collection of China, Third Institute of Oceanography, Ministry of Natural Resources, Xiamen, China, and given the accession number “Xia 20”. *Alternaria* sp. Xia 20 were statically incubated in medium at 25 °C for 31 days. The fermented culture was extracted with EtOAc three times to provide a crude extract. The extract was redissolved in MeOH and extracted with petroleum ether (PE) three times. The MeOH solution was evaporated under reduced pressure to obtain a defatted extract (25 g). The EtOAc extract was then subjected to column chromatography (CC) over silica gel using gradient CH2Cl2-MeOH (100:1→10:1) to provide four fractions (Fr.1−Fr.5). Fraction Fr.5 (5.7 g) was purified by CC on Sephadex LH-20 (MeOH) to yield AOH (155.0 mg).

AOH was isolated as a yellow powder. The molecular formula C14H10O5 was determined by the negative HR-ESI-MS at [M-H]- at m/z 257.1, indicating ten degrees of unsaturation. The 1H NMR data of AOH presented the diagnostic signals of four olefinic hydrogens at δH 7.19 (1H, d, J = 1.4 Hz, H-10), 6.67 (1H, d, J = 2.3 Hz, H-2), 6.59 (1H, d, J = 2.4 Hz, H-4), and 6.33 (1H, d, J = 1.5 Hz, H-8), and one methyl group at 2.68 (3H, s, H-11). The 13C NMR spectrum showed 14 carbon resonances consisting of one methyl (δC 25.2), four methines (δC 100.8, 101.6, 104.3, 117.5), and nine quaternary carbons (δC 97.4, 108.9, 138.1, 138.3, 152.6, 158.4, 164.1, 164.7, 165.4). Comparison of NMR data with those of the compound alternariol showed their structures were similar.

### 4.2. In Vitro Cell Models

The OVA-OTII system was previously reported [23]. Briefly, DCs were obtained from Rag1-/- mice and added to 96-well U-plates (1 × 10^5^ cells/well) with 5 µg/well of OVA (Sigma-Aldrich, St. Louis, MO, USA) at day 1. According to treatment with PBS or AOH, cells were divided into OVA + PBS and OVA + AOH groups. Purified naïve OTII CD4^+^ T cells (2 × 10^5^ cells/well) were incubated with DCs at day 0. For the iTreg differentiation, OVA-stimulated OTII CD4^+^ T cells were cultured with IL2 (5 ng/mL) and TGFβ (1 ng/mL) at day 0. Cells were cultured in RPMI1640 containing 10% FBS at 37 °C in an incubator. After 4 days, cells were harvested for antibody staining and analyzed by flow cytometer.

For BMDC and BMDM culture, bone marrow cells were obtained from the hind leg bone of mice, primary cells were cultured in RPMI1640 containing 10% FBS for 6 days with 10 ng/mL interleukin (IL)4 and 20 ng/mL GM-CSF for BMDC induction or 20 ng/mL GM-CSF for BMDM induction. Cells were seeded into 24-well plates and divided into PBS and AOH groups. 24 h later, cells were collected for flow cytometry to examine the effect of AOH on the maturation and function of BMDCs and BMDMs.

### 4.3. Antibodies

The following monoclonal antibodies were used for flow cytometry: Anti-CD4 (GK1.5), anti-CD8α (53–6.7), anti-CD25 (PC61.5), anti-CD44 (IM7), anti-CD62L (MEL-14), anti-CD69 (H1.2F3), anti-CD71(RI7217.1.4), anti-ICOS(C398.4A), anti-CD11b (M1/70), anti-CD11c (N418), anti-F4/80 (BM8), anti-Gr1 (RB6-8C5), anti-Foxp3 (FJK.16s), anti-MHCII(M5/114.15.2), anti-T-bet (4B10), anti-Eomes (Dan11mag), anti-TCRβ (H57-597), anti-IFNγ (XMG1.2), anti-TNFα (MP6-XT22), anti-IL-2 (JES6-5H4), anti-CD90.1 (Thy-1.1), anti-Siglec F (1RNM44N), anti-IL4 (11B11), anti-CD80 (16-10A1a), anti-CD86 (GL1), anti-B220 (RA3-6B2), anti-IgM (11/41), anti-c-Kit (2B8), anti-Ki6/7 (16A8), anti-CTLA4 (UC10-4B9), anti-Bcl2 (10C4), PE Annexin V Apoptosis Detection Kit I (559763). All the antibodies were purchased from BD Biosciences (San Jose, CA, USA), Biolegend (San Diego, CA, USA), or eBioscience (San Diego, CA, USA).

### 4.4. Mouse Model

In all the in vivo experiments, we treated AOH as a dose of 8 mg/kg according to our previous report for intervention of allergy using 8 mg/kg AME, a structurally analogous compound of AOH [22]. To detect immune homeostasis in mice after AOH treatment: C57BL/6J mice (4–5 weeks old) were randomly divided into two groups: PBS group and AOH group. Mice received 200 μL of PBS or AOH (8 mg/kg) via intragastric gavage (i.g.) once daily for 14 consecutive days. On day 15, mice were euthanized to collect immune-related organs, including LN, spleen, thymus, and bone marrow; T cell and B cell development; T cell activation; cytokine production were analyzed by flow cytometry. For the mouse model of lung injury, C57BL/6J mice (6–8 weeks old) were randomly divided into two groups: OVA^+^PBS group and OVA^+^AOH group. The mice were immunized with OVA/Alum (100 μg OVA) on days 0, 7, and 14. During immunization, the OVA+AOH group received 200 μL of AOH (8 mg/kg) orally for three consecutive days. On day 18, mice were intranasally challenged with 20 μL of 50 mg/mL OVA solution for four consecutive days and gavaged with PBS or AOH. Mice were dissected on day 22. Lung tissues were stained with hematoxylin-eosin (H&E), and immune cells in the LN and lungs were analyzed by flow cytometry.

The OT-II mice (#004194), C57BL/6J mice (#000664), and Rag1-/- mice (#003145) used in this study were purchased from Jackson Laboratories (USA). All animals were maintained in strict accordance with the Xiamen University Guide for the Care and Use of Laboratory Animals. The culture and operation protocols of the mice were approved by the Animal Research Ethics Committee of Xiamen University (Ethics No. XMULAC20230060, approved on 14 April 2023) and complied with the international regulations on the welfare and protection of laboratory animals.

### 4.5. In Vivo Cell Migration Assay

Two groups of cells were obtained using an in vitro OT-II (CD90.1 background) T cell differentiation system, OVA+PBS and OVA+AOH. On day 1, C57BL/6J mice (expressing a CD90.2 background) were divided into two groups. Each mouse was injected with CD4^+^ T cells (2 × 10^6^/mouse) by intravenous injection. On day 0, the mice were intranasally stimulated with OVA. On days 1 and 2, mice were dissected and analyzed for the proportion of CD4^+^ CD90.1^+^ cells in bronchoalveolar lavage fluid (BALF), lymph nodes (LNs), and lung immune cells.

### 4.6. Cell Acquisition

For collecting cells from BALF, an incision in the trachea of the mouse was made, and the lagging needle was inserted into the trachea. One milliliter of saline was inhaled with a syringe; the lagging needle was attached and the saline was injected into the lungs. The suction was repeated three times, and the above operation was repeated three times. The BALF was centrifuged to obtain cells.

For obtaining infiltrated immune cells from lungs, the lungs were mechanically dissected and digested at 37 °C for 60 min in RPMI with 10% FBS, 1% penicillin/streptomycin, 1 mg/mL collagenase Type 4 (Worthington Biochemical, Lakewood, NJ, USA), and 0.1 mg/mL DNase I (Sangon Biotech, Shanghai, China). The cells obtained by Percoll (Cytiva, Uppsala, Sweden) isolation were then used for flow cytometric staining and analysis.

### 4.7. Flow Cytometry

After obtaining the single cell suspension, the cell surface antibodies were added and incubated at 4 °C for 15 min. To analyze cytokine release, cells were stimulated (37 °C, 4 h) with PMA (YEASEN, Shanghai, China), Ionomycin (YEASEN, Shanghai, China), and GolgiPlug (BD Bioscience). After surface staining, cells were fixed and permeabilized using the fixation/permeabilization buffer kit (eBioscience, Boston, MA, USA) at 4 °C for 15 min. And then intracellular protein expression was assessed by staining cells at 4 °C for 1 h. To detect intracellular expression of transcription factors, cells were stained intranuclearly using a fixation/permeabilization buffer kit after labeling with surface antibodies. To observe cell proliferation, OTII CD4^+^ T cells (2 × 10^5^ cells/well, 96 wells) were pre-stained with 0.5 μM CFSE for 30 min at 37 °C and co-cultured with DC cells for 3.5 days. All flow cytometry data were collected on LSR Fortessa (BD Biosciences, Franklin Lakes, NJ, USA) and analyzed using FlowJo software 10 (Treestar (BD), Ashland, OR, USA).

### 4.8. Statistical Analysis

Statistical values were statistically processed using GraphPad Prism software 8.0, and HE staining results and immunofluorescence staining results were graphically processed using Case Viewer software 3.0. Statistical analysis was performed using *t*-test, “*”, *p* < 0.05, “**”, *p* < 0.001, “****”, *p* < 0.0001, ns indicates no significant differences, and the data were organized and summarized using Adobe Illustrator software v27(2023).

## 5. Conclusions

In conclusion, AOH isolated from marine fungi had significant anti-inflammatory activity via modulating the early activation and transcription process of T cells without targeting antigen-presenting cell function, inhibiting T cell proliferation, and reducing the release of inflammatory factors. With the inhibitory effects of inflammatory cytokine production and the regulation of T cell migration and infiltration, AOH showed an effective alleviating effect on OVA-induced lung injury and inflammation in mice. Therefore, as an active substance of marine origin, AOH is expected to provide a theoretical basis for the development of novel drugs for inflammatory and related immune diseases.

## Data Availability

The data used to support the findings of this study are included within the article. Further inquiries can be directed to the corresponding author.

## Supplementary Materials

The following supporting information can be downloaded at: https://www.mdpi.com/article/10.3390/md23030133/s1, Figure S1. NMR spectrum of AOH. Figure S2. The effect of AOH on CD4+ T cell numbers in OVA-OTII model in vitro. Figure S3. AOH does not affect immune cell development and homeostasis in steady stage. Figure S4. The impact of late-stage AOH intervention on lung-injured mice. Figure S5. AOH increases Treg differentiation in vitro and in vivo.
