# Supplementary material for: Marine-Derived Alternariol Suppresses Inflammation by Regulating T Cell Activation and Migration
Source: Mar Drugs. 2025 Mar 19;23(3):133. doi: 10.3390/md23030133 (PMC11944012; doi:10.3390/md23030133)

Marine-Derived Alternariol Suppresses Inflammation by Regulating T Cell Activation and Migration

Chenfeng Liu [1,†], Fudie Gu [2,†], Zhengbiao Zou [3], Fengli Wang [1], Dashuai Li [1], Jing Song [4], Yazhen Hong [4], Xuhui Wu [5], Xianwen Yang [3], Wen-Hsien Liu [4], Guangming Liu [2,6], Yu Zhou [4,*] and Qingmei Liu [2,*]

1 Department of Cell Biology, School of Life Science, Anhui Medical University, Hefei 230031, China; wfl19990316@163.com (F.W.); lds881008@163.com (D.L.)
2 Xiamen Key Laboratory of Marine Functional Food, Fujian Provincial Engineering Technology Research Center of Marine Functional Food, College of Ocean Food and Biological Engineering, Jimei University, Xiamen 361021, China; 15238875906@163.com (F.G.)
3 Hainan Academy of Medical Sciences, Hainan Medical University, Haikou 571199, China; yangxianwen@muhn.edu.cn (X.Y.)
4 State Key Laboratory of Cellular Stress Biology, School of Life Sciences, Faculty of Medicine and Life Sciences, Xiamen University, Xiamen 361102, China
5 School of Public Health, Xiamen University, Xiamen 361102, China
6 Faculty of Marine Biology, Xiamen Ocean Vocational College, Xiamen 361102, China
* Correspondence: yu_zhou1205@163.com (Y.Z.); liuqingmei@jmu.edu.cn (Q.L.); Tel.: +86-592-6180575 (Q.L.)
† The authors were contributed equally to this work.

Supplementary material

Figure S1. NMR spectrum of AOH.

A. ^{1}H NMR spectrum of AOH 1 in DMSO-d6. **B.** ^{13}C NMR spectrum of AOH 1 in DMSO-d6. C. A Xevo G2 Q-TOF mass spectrometer of AOH.

A

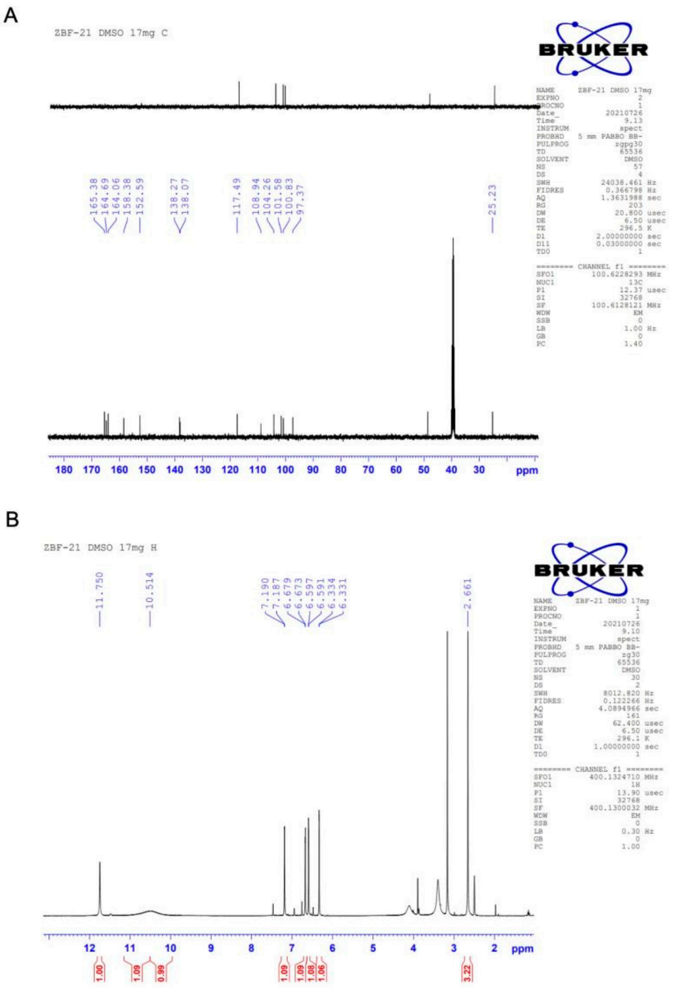

C

Elemental Composition Report

Tolerance = 20.0 PPM / DBE: min = -1.5, max = 50.0
Element prediction: Off
Number of isotope peaks used for i-FIT = 3

Monoisotopic Mass, Even Electron Ions
38 formula(e) evaluated with 0 results within limits (up to 50 closest results for each mass)
Elements Used:
C: 8-15 H: 0-30 N: 0-1 O: 0-10
ZBF-21-N 78 (0.318) Cm (72:78)
1: TOF MS ES-

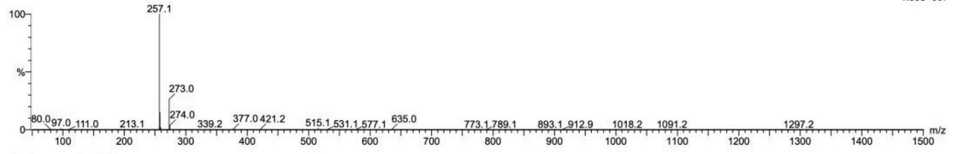

Minimum:	80.00				-1.5				
Maximum:	100.00		50.0	20.0	50.0				
Mass	RA	Calc. Mass	mDa	PPM	DBE	i-FIT	Norm	Conf(%)	Formula
257.0521	100.00	---							

Figure S2. The effect of AOH on CD4$^+$ T cell numbers in OVA-OTII model *in vitro*.

A. Absolute cell counts of CD4$^+$ T cells expressing CD69 and CD25 on Day1. **B.** Absolute cell counts of CD4$^+$ T cells expressing CD69 and CD25 on Day2. **C.** Absolute cell counts of CD4$^+$ T cells expressing CD69, CD25 and CD44 on Day3. Each symbol represents an independent biological replicate; the horizontal line represents the mean value (± s.e.m.). Statistical significance: ***， P < 0.001; ****， P < 0.0001. ns, no significance.

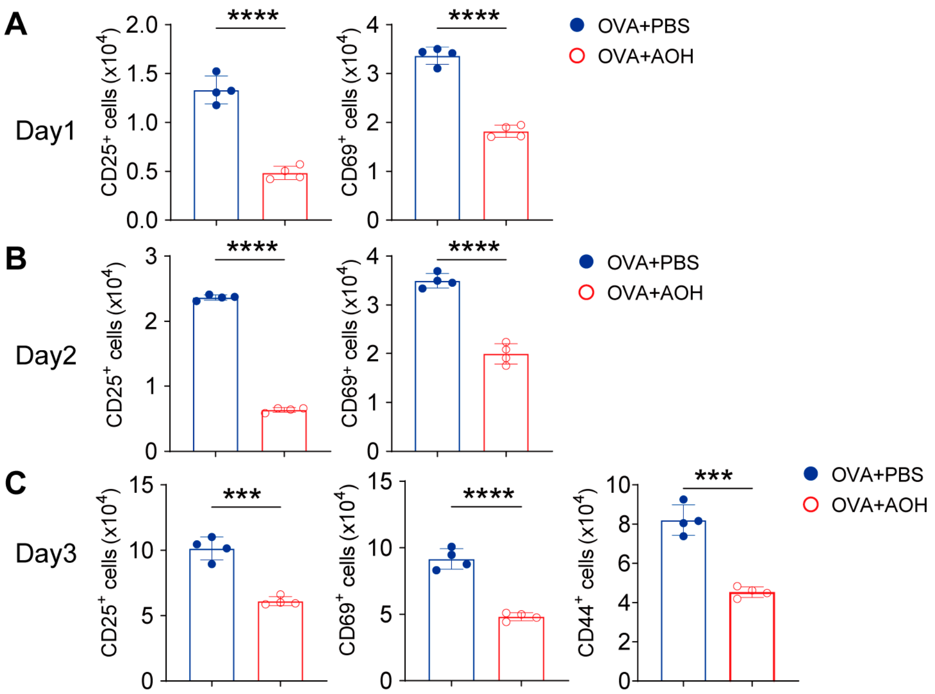

Figure S3. AOH does not affect immune cell development and homeostasis in steady stage.

A. Establishment of immune homeostasis model in mice. **B.** Absolute cell counts of immune organs. **C.** Development of T cells in the thymus. **D.** Absolute cell count of $CD8^+$ and $CD4^+$ T cells in the thymus. **E.** Development of B cells in the bone marrow. F. Proportion analysis of B cell subtypes. **G.** Percentage of progenitor and precursor B cells. **H.** Statistical analysis of progenitor and precursor B cells. **I.** Cytokine production of IL2 and IFNγ from $CD4^+$ T cells. **J.** Statistical graph of the proportion of IL2 and IFNγ released from $CD4^+$ T cells. **K.** Flow analysis of IFNγ produced by $CD8^+$ T cells.. **L.** Statistics of the proportion IFNγ released by $CD8^+$ T cells. Each symbol represents an independent biological replicate; the horizontal line represents the mean value (± s.e.m.). Statistical significance: *, $P<0.05$; **, $P<0.01$; ***, $P < 0.001$; ****, $P < 0.0001$. ns, no significance.

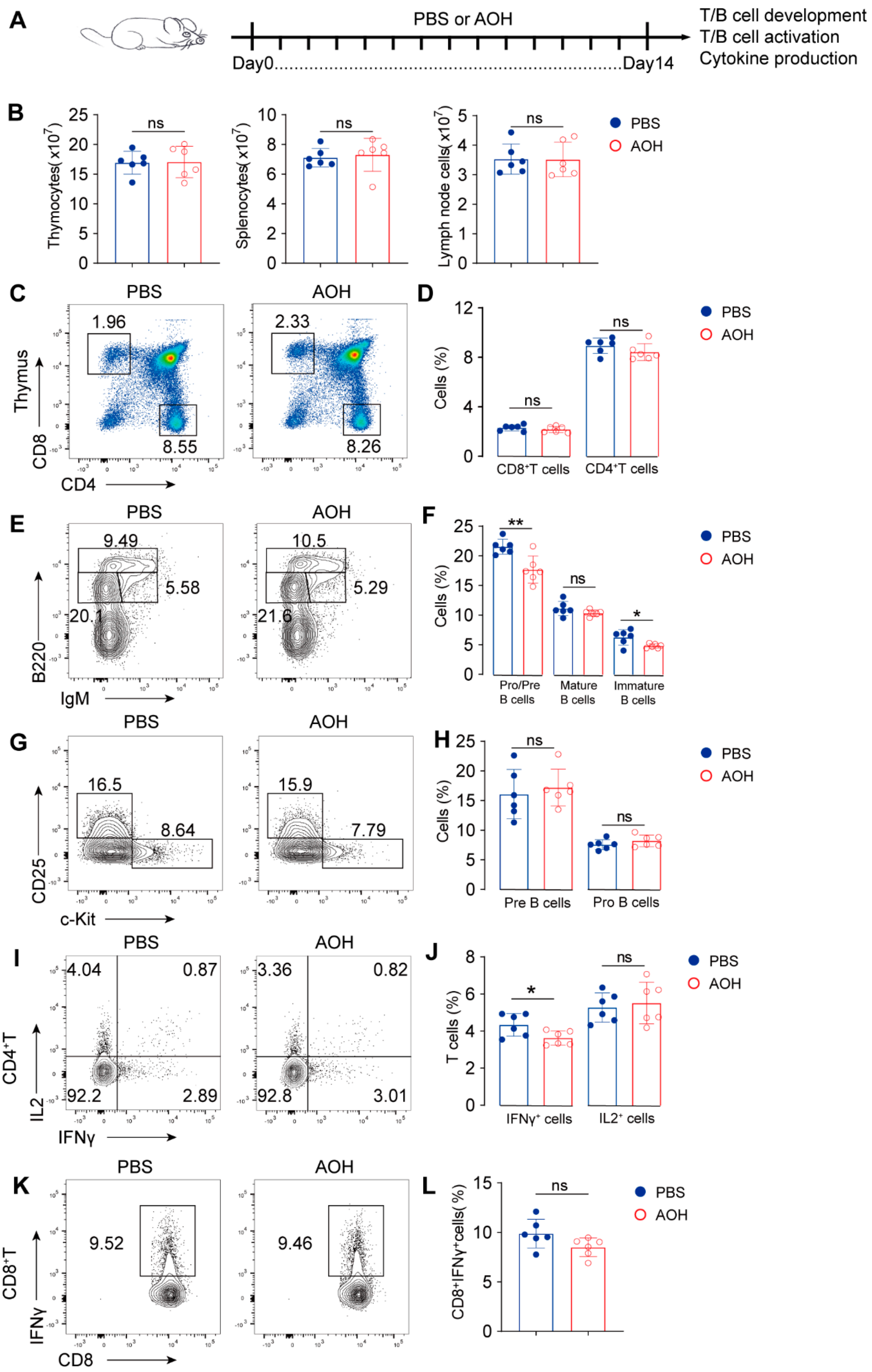
A
PBS or AOH
Day0
Day14
T/B cell development
T/B cell activation
Cytokine production
B
Thymocytes(x10^7)
Splenocytes(x10^7)
Lymph node cells(x10^7)
ns
PBS
AOH
C
PBS
AOH
Thymus
CD8
CD4
1.96
8.55
2.33
8.26
D
Cells (%)
CD8⁺T cells
CD4⁺T cells
E
B220
IgM
9.49
5.58
20.1
10.5
5.29
21.6
F
**
*
Pro/Pre B cells
Mature B cells
Immature B cells
G
CD25
c-Kit
16.5
8.64
15.9
7.79
H
Pre B cells
Pro B cells
I
CD4⁺T
IL2
IFNγ
4.04
0.87
92.2
2.89
3.36
0.82
92.8
3.01
J
T cells (%)
IFNγ⁺ cells
IL2⁺ cells
K
CD8⁺T
IFNγ
CD8
9.52
9.46
L
CD8⁺IFNγ⁺cells(%)

Figure S4. The impact of late-stage AOH intervention on lung-injured mice.

A. Experimental Model of Late-Stage AOH-Treated Lung Injury. **B.** Proportions of $CD4^+$ and $CD8^+$ T cells in LNs. **C.** Statistical analysis of the proportions and absolute cell counts of $CD4^+$ T cells and $CD8^+$ T cells in LNs. **D.** Flow cytometric analysis of IL4 and IFNγ release by $CD4^+$ T cells in LNs. **E.** Statistical analysis of the proportions and absolute cell counts of IL4 and IFNγ released by $CD4^+$ T cells in LNs. **F.** Flow cytometric analysis of IL4 and IFNγ released by $CD8^+$ T cells. **G.** Statistical analysis of the proportions and absolute cell counts of IL4 and IFNγ released by $CD8^+$ T cells in LNs. **H.** Flow cytometric analysis of $CD4^+$ T cells and $CD8^+$ T cells in lung. **I.** Proportions of T cells in lung. **J.** Flow cytometric analysis of IL4 and IFNγ release by $CD4^+$ T cells in lung. **K.** Statistical analysis of the proportions and absolute cell count of IL4 and IFNγ released by $CD4^+$ T cells in lung. **L.** Flow cytometric analysis of the proportions and absolute cell counts of IL4 and IFNγ released by $CD8^+$ T cells in lung. **M**. Statistical analysis of the proportions and absolute cell counts of IL4 and IFNγ released by $CD8^+$ T cells in lung. Each symbol represents an independent biological replicate; the horizontal line represents the mean value (± s.e.m.). Statistical significance: *，$P<0.05$; **，$P<0.01$; ***，$P < 0.001$; ****，$P < 0.0001$. ns, no significance.

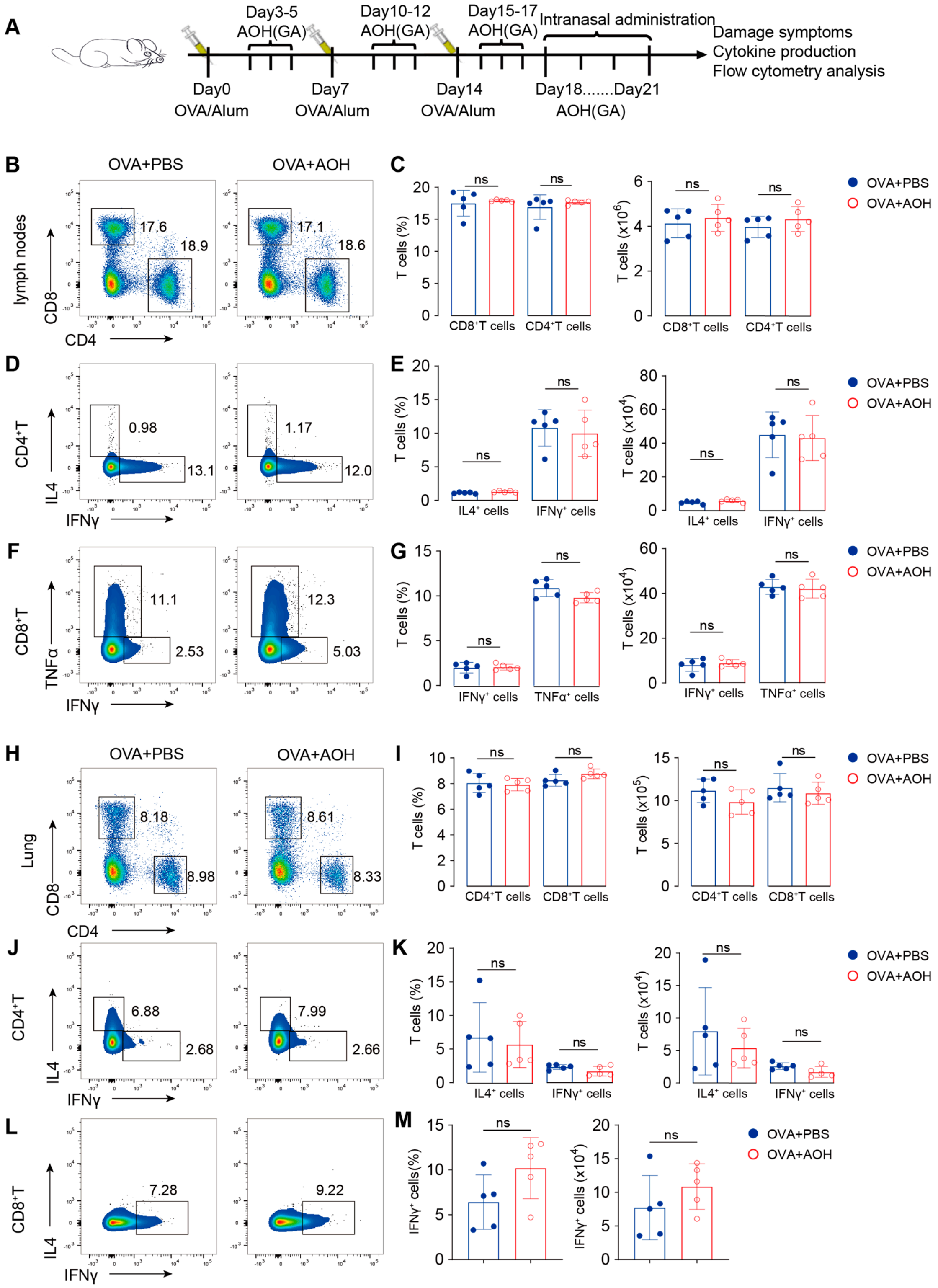

A
Day3-5
AOH(GA)
Day10-12
AOH(GA)
Day15-17
AOH(GA)
Intranasal administration
Damage symptoms
Cytokine production
Flow cytometry analysis
Day0
OVA/Alum
Day7
OVA/Alum
Day14
OVA/Alum
Day18.......Day21
AOH(GA)
B
OVA+PBS
OVA+AOH
lymph nodes
CD8
CD4
17.6
18.9
17.1
18.6
C
T cells (%)
T cells (x10^6)
ns
CD8+T cells
CD4+T cells
OVA+PBS
OVA+AOH
D
CD4+T
IL4
IFNγ
0.98
13.1
1.17
12.0
E
T cells (%)
T cells (x10^4)
IL4+ cells
IFNγ+ cells
F
CD8+T
TNFα
IFNγ
11.1
2.53
12.3
5.03
G
IFNγ+ cells
TNFα+ cells
H
OVA+PBS
OVA+AOH
Lung
CD8
CD4
8.18
8.98
8.61
8.33
I
T cells (%)
T cells (x10^5)
CD4+T cells
CD8+T cells
J
CD4+T
IL4
IFNγ
6.88
2.68
7.99
2.66
K
T cells (%)
T cells (x10^4)
IL4+ cells
IFNγ+ cells
L
CD8+T
IL4
IFNγ
7.28
9.22
M
IFNγ+ cells(%)
IFNγ+ cells (x10^4)

Figure S5. AOH increases Treg differentiation *in vitro* and *in vivo*.

A. Flow cytometric analysis and proportions of iTreg cell differentiation using OVA-OTII system plus IL2 and TGFβ cytokines *in vitro*. B. Flow cytometric analysis and proportions of Treg cell in lung. Statistical significance: *，P<0.05; **，P<0.01; ***，P < 0.001; ****，P < 0.0001. ns, no significance.

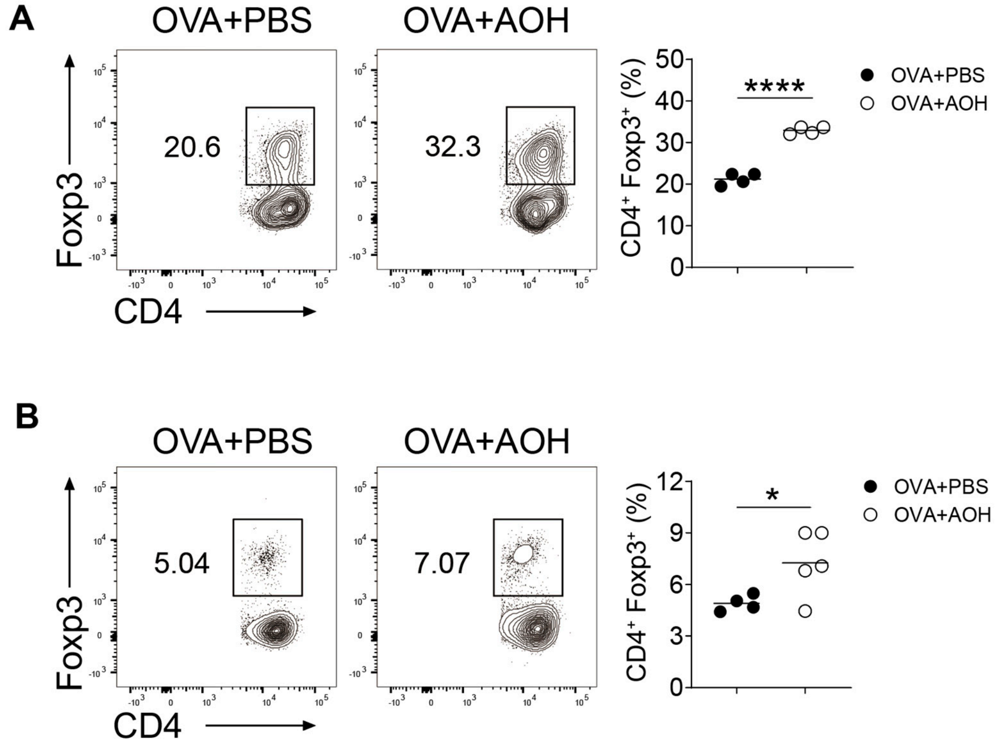

Supplement: Supplementary file 1 [file marinedrugs-23-00133-s001.zip › marinedrugs-3515807-supplementary.pdf]
